# Comparison of targeted next-generation sequencing for whole-genome sequencing of Hantaan orthohantavirus in *Apodemus agrarius* lung tissues

**DOI:** 10.1038/s41598-019-53043-2

**Published:** 2019-11-12

**Authors:** Jin Sun No, Won-Keun Kim, Seungchan Cho, Seung-Ho Lee, Jeong-Ah Kim, Daesang Lee, Dong Hyun Song, Se Hun Gu, Seong Tae Jeong, Michael R. Wiley, Gustavo Palacios, Jin-Won Song

**Affiliations:** 1 Department of Microbiology, College of Medicine, Korea University, Seoul, 02841 Republic of Korea; 2 Department of Microbiology, College of Medicine, Hallym University, Chuncheon, 24252 Republic of Korea; 3 Center for Medical Science Research, College of Medicine, Hallym University, Chuncheon, 24252 Republic of Korea; 4 4th R&D Institute, Agency for Defense Development, Daejeon, 34186 Republic of Korea; 5 The Center for Genome Sciences, U.S. Army Medical Research Institute of Infectious Diseases at Fort Detrick, Frederick, MD 21702 USA

**Keywords:** Genomics, Virology

## Abstract

Orthohantaviruses, negative-sense single-strand tripartite RNA viruses, are a global public health threat. In humans, orthohantavirus infection causes hemorrhagic fever with renal syndrome or hantavirus cardiopulmonary syndrome. Whole-genome sequencing of the virus helps in identification and characterization of emerging or re-emerging viruses. Next-generation sequencing (NGS) is a potent method to sequence the viral genome, using molecular enrichment methods, from clinical specimens containing low virus titers. Hence, a comparative study on the target enrichment NGS methods is required for whole-genome sequencing of orthohantavirus in clinical samples. In this study, we used the sequence-independent, single-primer amplification, target capture, and amplicon NGS for whole-genome sequencing of Hantaan orthohantavirus (HTNV) from rodent specimens. We analyzed the coverage of the HTNV genome based on the viral RNA copy number, which is quantified by real-time quantitative PCR. Target capture and amplicon NGS demonstrated a high coverage rate of HTNV in *Apodemus agrarius* lung tissues containing up to 10^3^–10^4^ copies/μL of HTNV RNA. Furthermore, the amplicon NGS showed a 10-fold (10^2^ copies/μL) higher sensitivity than the target capture NGS. This report provides useful insights into target enrichment NGS for whole-genome sequencing of orthohantaviruses without cultivating the viruses.

## Introduction

Orthohantaviruses belong to the family *Hantaviridae* of the order *Bunyavirales* and are enveloped negative-sense single-stranded RNA viruses^1,2^. The viral RNA genomes are segmented into large (L), medium (M), and small (S) segments. The tripartite segments encode an RNA-dependent RNA polymerase (RdRp), two envelope glycoproteins (Gn and Gc), and a nucleocapsid (N) protein. The absence of effective prevention and treatment strategies for hantavirus infections is a global public health threat^3,4^. In humans, infection from the Old World hantaviruses, e.g., Hantaan orthohantavirus (HTNV), Seoul orthohantavirus (SEOV), Dobrava-Belgrade orthohantavirus, and Puumala orthohantavirus, causes hemorrhagic fever with renal syndrome (HFRS), while infection from the New World hantaviruses, e.g., Sin Nombre orthohantavirus, Andes orthohantavirus, and New York virus, results in hantavirus cardiopulmonary syndrome^5^. Orthohantaviruses are transmitted to humans when viral infectious particles from the excreta of infected rodents are inhaled through the respiratory tract. HTNV, harbored by the striped field mouse (*Apodemus agrarius*), is an etiological agent for HFRS with fatality rates ranging between 1–15%^6,7^.

Next-generation sequencing (NGS) has been applied to various fields in virology, e.g., metagenomics of the virome, whole-genome sequencing, tracking the spread of virus, development of therapeutics, and identification of putative pathogens^8,9^. Whole-genome sequencing of emerging viruses is increasingly being used to identify and characterize viruses that pose a threat to human health^10–12^. However, the low abundance of viral particles in the hosts often presents an obstacle in virus identification. To diagnose and detect extremely low-titer viruses, several NGS methods have been developed, including enrichment of viral nucleic acids by using target-specific oligonucleotide probes, removing host genome sequences, purifying virus-like particles, and performing small-RNA deep sequencing^13–16^.

One of the most prominent techniques for random access to viral nucleic acids is sequence-independent, single-primer amplification (SISPA)^17,18^. Previously, it was used for sequencing HTNV isolates collected from HFRS endemic areas^19^. However, a significant limitation of whole-genome sequencing is the ultra-low number of copies of the target genome found in clinical or environmental specimens. To enrich the nucleic acids of interest, target-enrichment methods, including target capture or amplicon NGS, have been used to sequence viral genomes^20–22^. In target capture NGS, the samples are subjected to fragmentation, random reverse-transcription, ligation with barcoded library adapters, and polymerase chain reaction (PCR) amplification. These libraries are pooled and enriched using virus-specific probes. Recently, a novel target-enrichment deep-sequencing platform called “ViroFind”, which uses probes that cover the genome of 535 DNA or RNA viruses, was applied to the detection and analysis of viral populations in clinical samples^23^. Metsky *et al*. recovered Lassa virus genomes in low-titer clinical samples using multiple probe sets targeting 356 viral species, leading to the bio-surveillance in uncharacterized specimens^24^. To generate full-genome coverage, a tiling amplicon scheme is commonly used^25,26^. Previous studies have applied amplicon NGS to whole-genome sequencing of HTNV from patients and rodent hosts in HFRS endemic areas^27^. For this approach, the primer set was designed by amplifying short (150 nt) amplicons to maximize the recovery of virus genomic sequences. The whole-genome sequences of human- and rodent-derived HTNV revealed putative infection sites of HFRS patients by performing phylogeographic and epidemiological analyses. However, a comparison of the different target-enrichment NGS methods is yet to be conducted for the whole-genome sequencing of orthohantaviruses.

In this work, we prepared the RNA samples from *14A*. *agrarius* lung tissues and enriched the viral RNA using SISPA, target capture, and amplicon methods prior to the NGS library preparation. To evaluate three NGS methodologies, we analyzed and compared the depth of coverage and the recovery of virus genomes on a basis of the viral RNA copy number per μL.

## Results

### Sample selection for HTNV whole-genome sequencing

**Figure 1:**
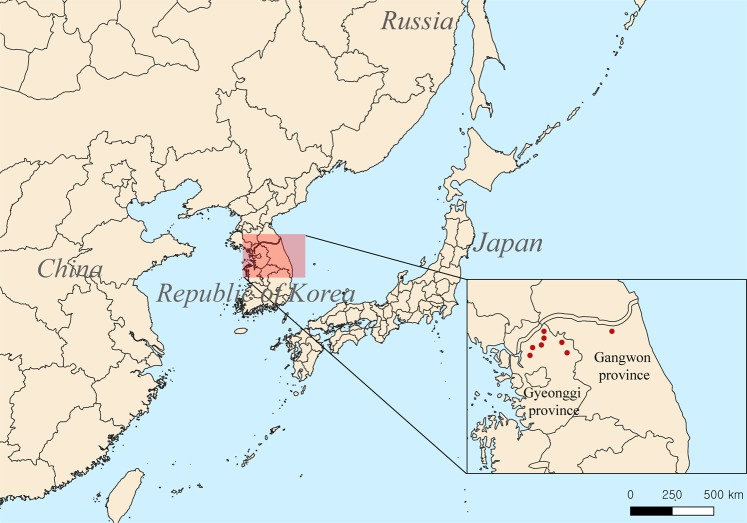
A geographic map of the trapping sites for *Apodemus agrarius* collected in the Republic of Korea. The trapping locations of rodents are shown in this study (red circles). Paju-si, Yeoncheon-gun, and Pocheon-si are included in Gyeonggi province. Yanggu-gun is localized in Gangwon province. We used the Quantum Geographical Information System (QGIS) software V.3.4.10. to create the geographic map.

A total of 161 *A*. *agrarius* were captured in HFRS-endemic areas including Gyeonggi and Gangwon provinces, Republic of Korea (ROK) between 2016 and 2017 (Fig. 1). Laboratory diagnosis examined the presence of anti-HTNV IgG and HTNV RNA by IFA and RT-PCR, respectively (Table 1, Supplementary Fig. 1). HTNV RNA loads was quantified on 14 sero- and RT-PCR-positive rodent lung tissues. The Ct values ranged from 20.8 to 32.8 regardless of the anti-HTNV IgG titer. The taxonomic identity of 14 *A*. *agrarius* was confirmed by sequencing the mitochondrial cytochrome *b* (Cyt *b*) gene. Phylogenetic analysis showed that 14 *A*. *agrarius* formed a genetic lineage with *A*. *agrarius* collected in the ROK (Supplementary Fig. 2).

**Table 1:** Selection and classification of the *Apodemus agrarius* lung tissues for whole-genome sequencing of Hantaan orthohantavirus (HTNV).

Viral RNA copy number	Strain	Location	HTNV		
			Anti-IgG Titer	Nested RT-PCR	Ct value
10^5^	Aa16-19	Pocheon	1:4,096	Pos	20.8
	Aa16-50	Yeoncheon	1:1,024	Pos	22.6
	Aa17-8	Paju	1:512*	Pos	21.4
	Aa17-49	Yeoncheon	1:128*	Pos	21.5
10^3^ to 10^4^	Aa16-181	Paju	1:256*	Pos	27.3
	Aa16-185	Paju	1:128*	Pos	25.1
	Aa17-48	Yeoncheon	1:512*	Pos	24.7
	Aa17-52	Yeoncheon	1:1,024	Pos	27.9
	Aa17-53	Yeoncheon	1:512	Pos	26.5
10^2^	Aa16-21	Pocheon	1:2,048	Pos	32.8
	Aa16-22	Pocheon	1:8,192	Pos	32.3
	Aa17-7	Paju	1:2,048	Pos	32.0
	Aa17-66	Pocheon	1:1,024	Pos	30.2
	Aa17-76	Pocheon	1:128*	Pos	32.1

^−^HTNV, Hantaan orthohantavirus; Aa, *Apodemus agrarius*; Nested RT-PCR, Nested Reverse Transcription-Polymerase Chain Reaction; Ct, Cycle threshold; Pos, Positive; IgG, Immunoglobulin G.

*In dead rodents, samples for IFA test was obtained from the heart.

Antibody titration was performed by using 2-fold dilutions starting at a diluted serum (1:32).

Nested RT-PCR was performed using HTNV specific primers.

### Determination of HTNV RNA copy number in *A*. *agrarius* lung tissues

HTNV RNA copy number was determined by generating a linear regression curve using a recombinant plasmid DNA, containing S segment of HTNV 76–118 (Supplementary Fig. 3). The coefficient of correlation (R^2^) value was 0.998 and the HTNV viral copy number of each sample was calculated. Aa16-19, Aa16-50, Aa17-8, and Aa17-49 showed Ct values ranging from 20.8–21.5 corresponding to 10^5^ copies/μL of viral RNA in the HTNV positive *A*. *agrarius* lung tissue. Aa16-181, Aa16-185, Aa17-48, Aa17-52, and Aa17-53 contained 10^3^ to 10^4^ copies/μL of HTNV RNA with 24.7–27.9 of Ct values. The Ct values of Aa16-21, Aa16-22, Aa17-7, Aa17-66, and Aa17-76 were 30.2–32.8, indicating that the rodents harbored 10^2^ copies/μL of HTNV RNA in the lung tissue.

### Whole-genome sequencing of HTNV using SISPA, target capture, and amplicon NGS

**Figure 2:**
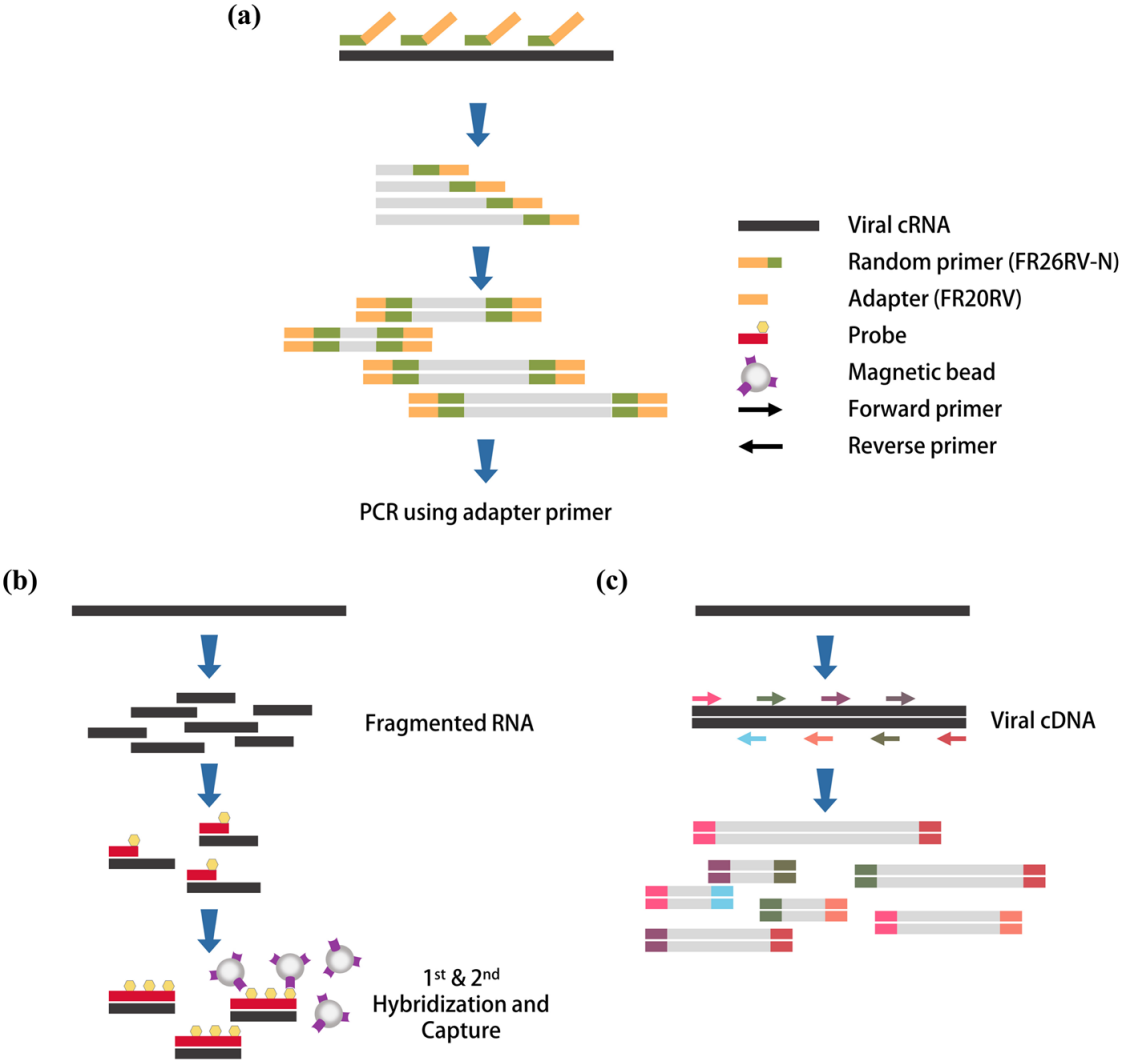
Description of different methods for next-generation sequencing (NGS) for Hantaan orthohantavirus (HTNV) in rodent lung tissues. Three methodologies of sample preparation were shown for NGS of HTNV. These methods amplified reads of viral genomes using different strategies. (**a**) For SISPA NGS method, RNA was reverse-transcribed using a random primer (FR26RV-N) and then cDNA was amplified using a single primer (FR20RV). (**b**) Target capture NGS captures viral genomes by using virus-specific probes. RNA samples were fragmented and then cDNA was synthesized using random hexamer. To enrich the target interest, first & second hybridization and capture were performed using virus-specific probes. (**c**) Amplicon NGS was applied to enrich viral genomes. Viral cDNA was amplified using HTNV-specific multiple primer mixture.

**Figure 3:**
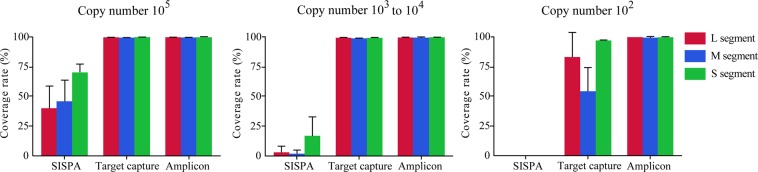
Coverage rate of consensus sequences for Hantaan orthohantavirus (HTNV) by viral copy number. The percentage coverage rate of consensus sequences by viral copy number among the three next-generation sequencing methods. Coverage rate was calculated by matching the consensus sequences with the sequence of HTNV 76–118 strain (GenBank accession number, NC_005222, NC_005219, NC_005218).

To obtain the whole-genome sequence of HTNV, all samples were sequenced in the MiSeq using SISPA, target capture, and amplicon NGS (Fig. 2). SISPA NGS showed the lowest coverage of HTNV tripartite genome for the lung tissues containing HTNV RNA of 10^5^ copies/μL; 40%, 45%, and 75% for L, M, and S segments, respectively, compared to full-length nucleotides of the prototype HTNV 76–118 (Fig. 3). The coverage of HTNV genomic sequence remarkably decreased as the copy number of viral RNA was low (10^3^–10^4^ to 10^2^ copies/μL). Using target capture NGS, nearly complete genome sequence of HTNV was recovered in the samples carrying more than 10^3^ copies/μL of HTNV RNA (L segment, 99.6%; M segment, 99.1%; S segment, 99.6%). In the samples with low viral RNA copy number of 10^2^ copies/μL, decreases of the coverage rate were observed for L (84.9%) and M (69.4%) segments, while the coverage of S segment was slightly reduced (96.8%). Finally, amplicon NGS recovered nearly whole-genome sequences of HTNV from 10^5^ to 10^2^ copies/μL of the viral RNA copy numbers.

### Comparison for coverage and depth of HTNV whole-genome sequences based on the different NGS methods

**Figure 4:**
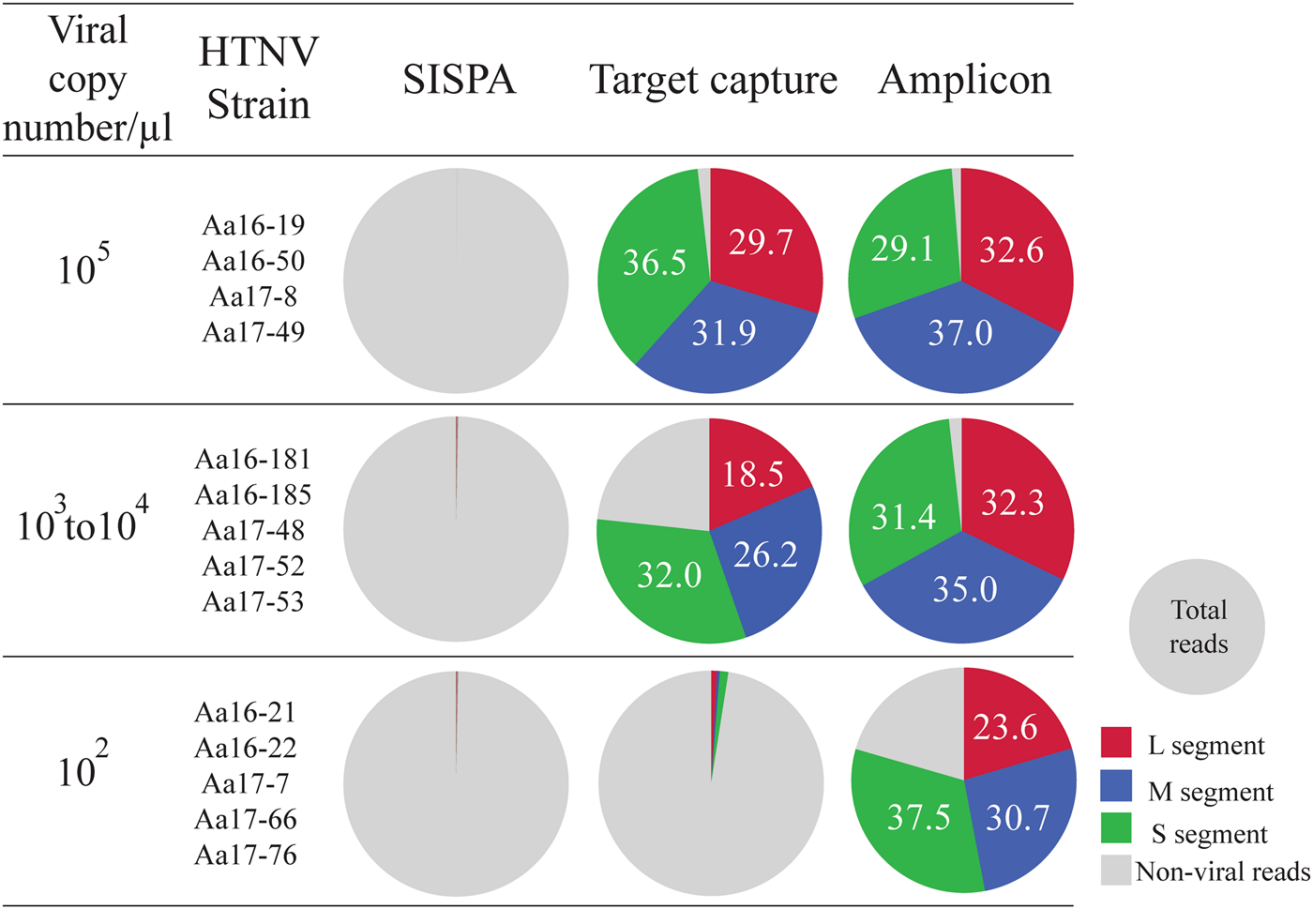
The composition of Hantaan orthohantavirus (HTNV) genomic reads in the total reads generated by three next-generation sequencing methods. The composition of viral reads mapped to HTNV was exhibited in the total number of reads. The total reads were produced by performing SISPA, target capture, and amplicon NGS. These reads were mapped to HTNV 76–118 tripartite genomes (GenBank accession number: L segment, NC_005222; M segment, NC_005219; S segment, NC_005218). A circle represents for total reads obtained by SISPA, target capture, and amplicon NGS methods. Red, Blue, and Green colors indicate the composition of viral reads for HTNV L, M and S segments, respectively, over the total reads. Gray color indicates non-viral reads in the total reads. The HTNV reads were shown as a percentage (%) evaluated by the ratio of viral reads over the total reads.

The composition of reads mapped to HTNV genomes was shown in the total reads generated by three NGS methods (Fig. 4). In the SISPA NGS, an average of 673,832 total reads were observed (Supplementary Table 1). The average number of viral sequence reads were 22 (0.003%) for L segment, 16 (0.002%) for M segment, and 33 (0.005%) for S segment. The average depth of coverage was scarcely found; one for M segment and three for S segment. In the target capture NGS, 443,731 (26.2%), 480,973 (28.4%), and 552,172 (32.6%) reads out of a total of 1,692,164 reads mapped to the reference sequence for the HTNV L, M, and S segments, respectively (Supplementary Table 2). The total read numbers and depth of coverage for HTNV were decreased corresponding to the 10^5^ to 10^2^ copies/μL viral RNA copy numbers. Lastly, in amplicon NGS we observed 438,666 (30.3%), 444,437 (30.7%), and 468,114 (32.3%) reads out of a total of 1,448,091 reads for HTNV L, M, and S segments, respectively (Supplementary Table 3). The viral read counts and depth of coverage were comparable at the HTNV RNA copy numbers of 10^5^ and 10^3^ to 10^4^ copies/μL. However, the read counts and depth of coverage for HTNV L and M segments were reduced at viral RNA copy numbers of 10^2^ copies/μL except for the S segment.

### Phylogenetic analysis

**Figure 5:**
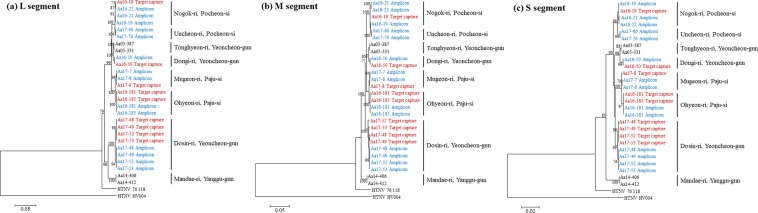
Phylogenetic analysis of Hantaan orthohantavirus (HTNV) whole-genome sequences using target capture and amplicon next-generation sequencing (NGS). Phylogenetic trees were generated by the ML method using the TN93 + G, T92 + G, and T92 + G model based on (**a**) L segment (1–6530 nt), (**b**) M segment (1–3616 nt), Red characters indicate and (**c**) S segment (1–1696 nt), respectively. The numbers at each node are bootstrap probabilities, as determined for 1,000 iterations. Sequences obtained in target capture NGS were indicated in red. Sequences obtained in amplicon NGS were indicated in blue. Other hantaan orthohantaviruses were used as outgroups. HTNV Aa03–387 (L segment, KT934958; M segment, KT934992; S segment, KT935026), HTNV Aa05-331 (L segment, KT934962; M segment, KT934996; S segment, KT935030), HTNV Aa14-406 (L segment, KT934985; M segment, KT935019; S segment, KT935053), HTNV Aa14-412 (L segment, KT934987; M segment, KT935021; S segment, KT935055), HTNV 76-118 (L segment, NC_005222; M segment, NC_00519; S segment, NC_005218), and HTNV HV004 (L segment, JQ083393; M segment, JQ083394; S segment, JQ093395).

To validate the whole-genome sequences of HTNV obtained by target enrichment NGS methods, phylogenetic trees were generated by maximum likelihood (ML) method (Fig. 5). The phylogenies of HTNV L, M, and S segments from Aa16-181, Aa16-185, and Aa17-8 formed a genetic lineage with the strains collected from Paju-si. The phylogenetic pattern of HTNV tripartite genome from Aa16-19 shared a common ancestor with the HTNV strain in Pocheon-si. Phylogenetically, HTNV L, M, and S segments from Aa16-50 grouped to the HTNV strains from Tonghyeon-ri, Yeoncheon-gun, while Aa17-48, Aa17-49, Aa17-52, and Aa17-53 grouped to the HTNV strains from Dosin-ri, Yeoncheon-gun.

## Discussion

Whole-genome sequences of viruses play a critical role in the rapid identification and tracking of the infection source during disease outbreaks. To take advantage of the whole-genome sequence, various NGS methodologies are utilized for the rapid and robust virus genomic sequencing prior to culturing of virus. SISPA NGS was used for characterizing RNA and DNA viruses including Astrovirus, Parvovirus, and Rotavirus^28–30^. Target capture NGS was implemented as an important tool for the genome sequencing of ZIKV (Zika virus), Ebola virus and chikungunya virus^21,31,32^. The complete genome sequences of ZIKV, Respiratory syncytial virus and HTNV were obtained by amplicon NGS^21,27,33^. However, different NGS methods remain to be comparatively evaluated for optimal whole-genome sequencing of specimens containing low viral RNA loads.

This study describes different NGS methodologies, SISPA, target capture, and amplicon NGS, for the whole-genome sequencing of HTNV directly from the host rodent lung tissues without the need for cultivating virus particles. To compare the robustness of individual methods, the whole-genome sequencing of HTNV was performed by using tissue specimens containing different viral RNA loads ranging from 10^2^ to 10^5^ copies/μL. Although SISPA is a representative method to gain whole-genome sequence of viruses via high-throughput sequencing, poor genome coverage rates of HTNV, even in samples containing highest RNA copy number (10^5^) per μL, were observed in this study. The limitation for SISPA NGS was low sensitivity for obtaining the viral whole-genome sequence from the specimen containing low titer of the virus or non-isolated samples. On the basis of whole-genome sequencing of HTNV, target capture NGS enabled recovery of nearly complete genome sequences of HTNV from 10^5^ to 10^3^–10^4^ copies/μL of HTNV RNA loads. At the HTNV RNA loads of 10^2^ copies/μL, coverage rates of HTNV genome sequences remarkably decreased for L and M segments. The coverage of HTNV S segments showed over 95% recovery at this level due to short length of the genome. The whole-genome sequences of HTNV by amplicon NGS were completely recovered from 10^5^ to 10^2^ copies/μL of HTNV RNA loads. Several studies that used a tiling amplicon scheme showed reliable coverage rate (>95%) of the whole viral genome by long amplicons (1–2.5 kb in length) using NGS^25,34,35^. However, with lower virus abundance in tissue, long fragments reduced the coverage rate of genome sequence. To ensure high coverage at low copy number of HTNV, we developed the amplicon NGS using specific primer sets which generate few hundred-length short amplicons. This methodology is robust and efficient for acquiring the whole-genome sequence of HTNV and SEOV from patients and rodent samples^22,27^. In this study, the capacity of amplicon NGS was delineated up to 10^2^ copies/μL of HTNV RNA loads.

Obtaining whole-genome sequences in clinical samples or in samples with low virus titers is problematic. Worobey *et al*. employed a “jackhammering” approach to the detection of target RNA molecules in degraded and low-titer samples^36^. This approach used primer pools to acquire genomes by generating a large number of small amplicons of the target genome. High-copy templates of the dengue virus genome were fully acquired with a few amplicons, whereas low-copy viral templates could be recovered using 10 overlapping amplicons^37^. These results demonstrate that short amplicons are easier to generate than long amplicons in low-copy viral specimens. The usage of multiplex primer sets to generate amplicons is an efficient strategy for obtaining viral genomes in low-titer specimens without the need for cultivating the viruses. However, amplicon-based methods introduce PCR artifacts due to the presence of Taq DNA polymerase through a number of PCR cycles. In addition, multiplex primers could generate amplicons for unrecognized unexpected variants^38^. In this study, multiplex primer sets facilitated the whole-genome sequencing of HTNV due to high nucleotide homology among virus strains; however, the efficiency of amplicon sequencing of viruses with low nucleotide homology should be evaluated.

In conclusion, we evaluated different methodologies for target enrichment NGS coverage for the complete genome sequence of HTNV from the rodent lung tissues. The amplicon NGS is the most robust method to recover nearly whole-genome sequences of HTNV from the specimen containing up to 10^2^ copies/μL of HTNV RNA loads. Thus, this study provides useful insights into target enrichment NGS for the rapid identification and characterization of orthohantaviruses during endemic outbreaks.

## Methods

### Ethics statement

This study was approved in accordance with the ethical guidelines for the Korea University Institutional Animal Care and Use Committee (KU-IACUC), Korea University. Rodents trapping at US military training sites and installations was approved by US Forces Korea in accordance with regulation 40-1 (Prevention, Surveillance, and Treatment of Hemorrhagic Fever with Renal Syndrome). Rodents were euthanized by cardiac puncture under isoflurane anesthesia and the tissues were collected in accordance with the procedures approved by KU-IACUC (#2016-0049) protocol. All experiments were conducted in the biosafety level 3 facility.

### Sample selection

Rodents (*A*. *agrarius*, *Mus musculus*, and *Myodes regulus*) were captured using live-capture Sherman traps (H.B. Sherman, Tallahassee, FL, USA) in Gyeonggi province, ROK between 2016 and 2017. Rodent species were identified by their morphology. Sera, lung, spleen, kidney, and liver tissues of the rodents were collected aseptically and frozen at −80 °C until use. For the whole-genomic sequencing of HTNV, the sera of the rodents were used for detecting the anti-HTNV immunoglobulin G (IgG) using indirect immunofluorescence assay (IFA) test. HTNV RNA was detected using HTNV-specific primers by RT-PCR. Lung tissues, which were positive in both sera anti-HTNV IgG and RT-PCR, from the rodents were selected and quantified for the HTNV genome copy number.

### Indirect immunofluorescence antibody test

Rodent sera were diluted 1:32 in phosphate-buffered saline and examined for IgG against HTNV. The diluted sera were added to acetone-fixed HTNV-infected Vero E6 cells and the cells were incubated at 37 °C for 30 min. The cells were washed and treated with fluorescein isothiocyanate-conjugated anti-mouse IgG (ICN Pharmaceuticals, Laval, Quebec, Canada), and the cells were further incubated at 37 °C for 30 min. The cells were washed, and virus-specific fluorescence was examined by using a fluorescent microscope (Axio Scope, Zeiss, Berlin, Germany).

### Mitochondrial DNA (mtDNA) analysis

Total DNA was extracted from liver tissues using TRIzol reagent solution (Life Technologies, Carlsbad, CA, USA). Rodent species was confirmed by performing mitochondrial Cyt *b* gene-specific PCR^39^.

### RNA extraction and cDNA synthesis

Total RNA was extracted from the lung tissues of *A*. *agrarius* using a Hybrid R Kit (GeneAll Biotechnology, Seoul, ROK) according to the manufacturer’s specifications. cDNA was synthesized using a High Capacity RNA-to-cDNA Kit (Applied Biosystems Inc., Carlsbad, CA, USA) with random hexamers or OSM55 (5′-TAGTAGTAGACTCC-3′) primer.

### Nested RT-PCR

Nested PCR was performed in a 25 μL reaction mixture containing 0.1 mM dNTP Mix, 0.625 units TaKaRa Ex Taq polymerase (Takara, Shiga, Japan), 0.4 µM of each primer, and 1.5 µL template. Oligonucleotide primer sequences for the nested PCR were G2F1 (outer): 5′-TGGGCTGCAAGTGC-3′, G2-2 (outer): 5′-ACATGCTGTACAGCCTGTGCC-3′, G2-1 (inner): 5′-TGGGCTGCAAGTGCATCAGAG-3′, and G2-4 (inner): 5′-ATGGATTACAACCCCAGCTCG-3′ for the M segment^40^. The PCR conditions were: initial denaturation at 94 °C for 5 min, followed by 6 cycles of denaturation at 94 °C for 30 s, annealing at 37 °C for 30 s, elongation at 72 °C for 1 min, followed by 32 cycles of denaturation at 94 °C for 30 s, annealing at 42 °C for 30 s, elongation at 72 °C for 1 min. Additionally, final elongation was done at 72 °C for 5 min. PCR products were extracted using a PCR Purification Kit (Cosmo Genetech, Seoul, ROK), and DNA sequencing performed in an Automatic Sequencer, ABI 3730XL DNA Analyzer (Applied Biosystems, USA).

### Real-time quantitative PCR

RT-qPCR was performed using SYBR Green PCR Master mix (Applied Biosystems) on a Quantstudio 5 Flex Real-Time PCR System (Applied Biosystems). The primer sequences included a forward primer; 5′-TTATTGTGCTCTTCATGGTTGC-3′ and a reverse primer; 5′-CATCCCCTAAGTGGAAGTTGTC-3′ for HTNV S segment^41^. The PCR conditions were: 95 °C for 10 min, followed by 40 cycles of 15 s at 95 °C and 1 min at 60 °C.

### Quantitation of HTNV genome copy

Viral RNA was extracted from HTNV 76–118, and cDNA was synthesized using a High Capacity RNA-to-cDNA kit (Applied Biosystems). PCR was performed in a 25 µL reaction mixture, containing 0.1 mM dNTP Mix, 0.625 units TaKaRa Ex Taq polymerase (Takara, Shiga, Japan), 0.4 µM of each primer, and 1.5 µL cDNA. Primer sequences were HTN-S-1127F (5′–CCTACCTCAGAAGGACACAATC–3′) and HTN-S-1437R (5′–ATGTTCCCATGCCCTGATATAC–3′). The amplified products were cloned into the pSTBlue-1 AccepTor^TM^ vector (EMD Millipore Corp., Billerica, MA, USA) using T-A cloning technique with the DNA Ligation Kit (TaKaRa). The recombinant plasmid DNA was isolated using the Hybrid-Q Plasmid Preparation Kit (GeneAll Biotechnology). The concentration of recombinant plasmid DNA was measured by UV absorbance at 260 nm and 280 nm using Nano drop. Serial dilutions of the recombinant plasmid DNA standards ranging from 1 × 10^10^ to 1 × 10^3^ copies/µL were amplified using SYBR Green PCR Master mix (Applied Biosystems) on a Quantstudio 5 Flex Real-Time PCR System (Applied Biosystems). The primer sequences and PCR conditions were identical to the ones used in RT-PCR. The copy number of plasmids per microgram of DNA was calculated using the total number of nucleotides in the plasmid using a described previously formula^42^.

$$[{\rm{copy}}\,{\rm{number}}\,{\rm{of}}\,{\rm{plasmid}}=({\rm{amount}}\,{\rm{of}}\,{\rm{plasmid}}\,({\rm{ng}})\times 6.022\times {10}^{23})/({\rm{length}}\,{\rm{of}}\,{\rm{plasmid}}\times 1\times {10}^{9}\times 660)]$$

### Sequence-independent single-primer amplification

cDNA was generated from total RNA using FR26RV-N (5′-GCCGGAGCTCTGCAGATATCNNNNNN-3′). The reaction was performed in a 20 μL reaction mixture containing 7 μL total RNA, 2 μL 10 pM of primer, 2 μL 5X First strand buffer, 100 mM dithiothreitol, 25 mM MgCl_2_, 10 mM dNTPs, 0.5 μL RNaseOUT, and 0.5 μL Superscript III RTase (Life Technologies, Carlsbad, CA) in a Proplex thermocycler (Life technologies). The PCR conditions were: 25 °C for 10 min, 50 °C for 50 min, and 85 °C for 10 min. Double-stranded (ds) cDNA was synthesized using 0.2 units Klenow 3′ → 5′ exo DNA polymerase (Enzynomics, Daejeon, ROK) and1 μL RNaseH (Invitrogen, San Diego, CA). The Klenow reaction mixture was incubated at 37 °C for 1 h and 75 °C for 15 min. The ds cDNA was purified using MinElute PCR purification kit (Cat No. 28004, Qiagen, Hilden, Germany). Using the FR20RV (5′-GCCGGAGCTCTGCAGATATC-3′) primer, ds cDNA was amplified in a 50 μL reaction mixture containing 10 μL ds cDNA template, 10 pM primer, and 2X My Taq Red (Bioline, Taunton, MA). PCR conditions were: Initial denaturation at 98 °C for 30 s, followed by 38 cycles of denaturation at 98 °C for 10 s, annealing at 54 °C for 20 s, and elongation at 72 °C for 45 s.

### Target capture-based enrichment

The custom-made probes (kindly provided by Dr. Gary Schroth and Dr. Stephen Gross, Illumina, San Diego, CA, USA) were designed to cover the entire HTNV tripartite genome based on the HTNV 76–118 (L segment, NC_005222; M segment, NC_005219; S segment, NC_005218) and ROKA 14-11 (L segment, KU207199; M segment, KU207203; S segment, KU207207) strains. The sequence of primers was shown in the Supplementary Data 1. Extracted RNA was fragmented at 94 °C for 10 s and each sample were enriched separately using a quarter of the reagents specified in the manufacturer’s protocol of the TruSeq RNA Access library preparation kit (Illumina). Samples were barcoded, pooled, and sequenced using the MiSeq reagent kit v2 (Illumina) on an Illumina MiSeq with a minimum of 2 × 150-bp reads.

### Amplicon-based enrichment

cDNA was amplified using the primer mixture and Solg™ 2X Uh-Taq PCR Smart mix (Solgent, Daejeon, ROK) according to the manufacturer’s instruction. PCR condition and sequences of primer sets were as described previously^27^.

### Library preparation for NGS

Libraries were prepared using TruSeq Nano DNA LT sample preparation kit (Illumina according to the manufacturer’s instructions. Double stranded cDNA was sheared using a M220 focused ultra-sonicator (Covaris, Woburn, MA, USA). The ds cDNA was size-selected, followed by evaluating the quality and concentration of the samples using an Agilent DNA 1000 chip kit or a high-sensitivity DNA chip kit on a bio-analyzer (Agilent Technologies, Santa Clara, CA, USA). The libraries were A-tailed, ligated with double indexes and adaptors, and enriched by PCR. The libraries were quantified using the Library Quantification Kit (KAPA Biosystems, Wilmington, MA, USA) on a Quantstudio 5 Flex Real-Time PCR System (Applied Biosystems). NGS sequencing was performed on a MiSeq benchtop sequencer (Illumina) with 2 × 150 base pairs using a MiSeq reagent kit v2 (Illumina).

### NGS data analysis

Adaptor and index sequences of reads were trimmed, and low-quality sequences were filtered using CLC Genomics Workbench version 7.5.2 (CLC Bio, Cambridge, MA). The tripartite genome sequence of HTNV 76-118 was used in a reference mapping method. The read mapping to the reference genome sequence and the extraction of consensus sequences were performed. The genomic sequences of HTNV strains were deposited in GenBank (Supplementary Table 4).

### Phylogenetic analysis

Multiple sequences of HTNV were aligned using MUSCLE algorithm in MEGA 6.0^43^. The best fit substitution model was determined for HTNV L (1–6530 nt), M (1–3616 nt) and S segments (1–1696 nt), which were TN93 + G, T92 + G, and T92 + G, respectively. ML method was employed to generate the phylogenetic trees using MEGA 6.0. Topologies were assessed by bootstrap analysis of 1000 iterations.

## Supplementary information

Supplementary information

Supplementary data 1

## Data Availability

Raw fastq reads have been deposited in the NCBI SRA database (Supplementary Information).
