# Supplementary material for: Comparison of targeted next-generation sequencing for whole-genome sequencing of Hantaan orthohantavirus in Apodemus agrarius lung tissues
Source: Sci Rep. 2019 Nov 12;9:16631. doi: 10.1038/s41598-019-53043-2 (PMC6851128; doi:10.1038/s41598-019-53043-2)
Supplement: Supplementary file 1 — Supplementary information [file 41598_2019_53043_MOESM1_ESM.pdf]

Supplementary information

Comparison of targeted next-generation sequencing for whole-genome sequencing of Hantaan orthohantavirus in *Apodemus agrarius* lung tissues

Jin Sun No[1,†], Won-Keun Kim[2,3†], Seungchan Cho[1,†], Seung-Ho Lee[1], Jeong-Ah Kim[1], Daesang Lee[4], Dong Hyun Song[4], Se Hun Gu[4], Seong Tae Jeong[4], Michael R. Wiley[5], Gustavo Palacios[5], and Jin-Won Song[1,*]

[1]Department of Microbiology, College of Medicine, Korea University, Seoul 02841, Republic of Korea, [2]Department of Microbiology, College of Medicine, Hallym University, Chuncheon 24252, Republic of Korea, [3]Center for Medical Science Research, College of Medicine, Hallym University, Chuncheon 24252, Republic of Korea, [4]4th R&D Institute, Agency for Defense Development, Daejeon 34186, Republic of Korea, [5]The Center for Genome Sciences, U.S. Army Medical Research Institute of Infectious Diseases at Fort Detrick, MD 21702, USA

†These authors contributed equally to this study.

*Correspondence: jwsong@korea.ac.kr

Corresponding author: Jin-Won Song, M.D., Ph.D.

Korea University

College of Medicine

Department of Microbiology

Inchon-ro 73, Seongbuk-gu, Seoul 02841, Republic of Korea

Tel: 82-2-920-6353

Fax: 82-2-923-3645

E-mail: jwsong@korea.ac.kr

Supplementary figure legend

Supplementary Fig 1. Detection of IgG antibodies to Hantaan orthohantavirus (HTNV) in rodent sera by indirect immunofluorescence assay (IFA).

HTNV-infected Vero E6 cells were placed and fixed on a slides. The rodent sera (1:32 dilution) and FITC-conjugated anti-mouse IgG were added for detecting anti-HTNV IgG by IFA. The images were taken at using a 400× objective.

Supplementary Fig 2. A **phylogenetic tree based on the mitochondrial cytochrome b gene of** ***Apodemus spp.***

HTNV-infected *A. agrarius* was confirmed by PCR for mitochondrial cytochrome b gene (1,041 nt). A phylogenetic tree was generated by the maximum likelihood method. The bootstrap support values more than 70% are shown at the nodes.

Supplementary Fig 3. A standard curve for the quantitation of Hantaan orthohantavirus (HTNV) loads in the rodent tissue.

Standard curve obtained with serial 10-fold dilutions of HTNV S segment recombinant plasmid DNA, ranging from 1×10^{10} to 1×10^{3} copies/μL. Each point represents the mean threshold cycle (Ct) value obtained from triplicates. The slope of the plasmid DNA standard curve is 3.218, with correlation coefficient (R2) of 0.998.

Supplementary Fig 1.

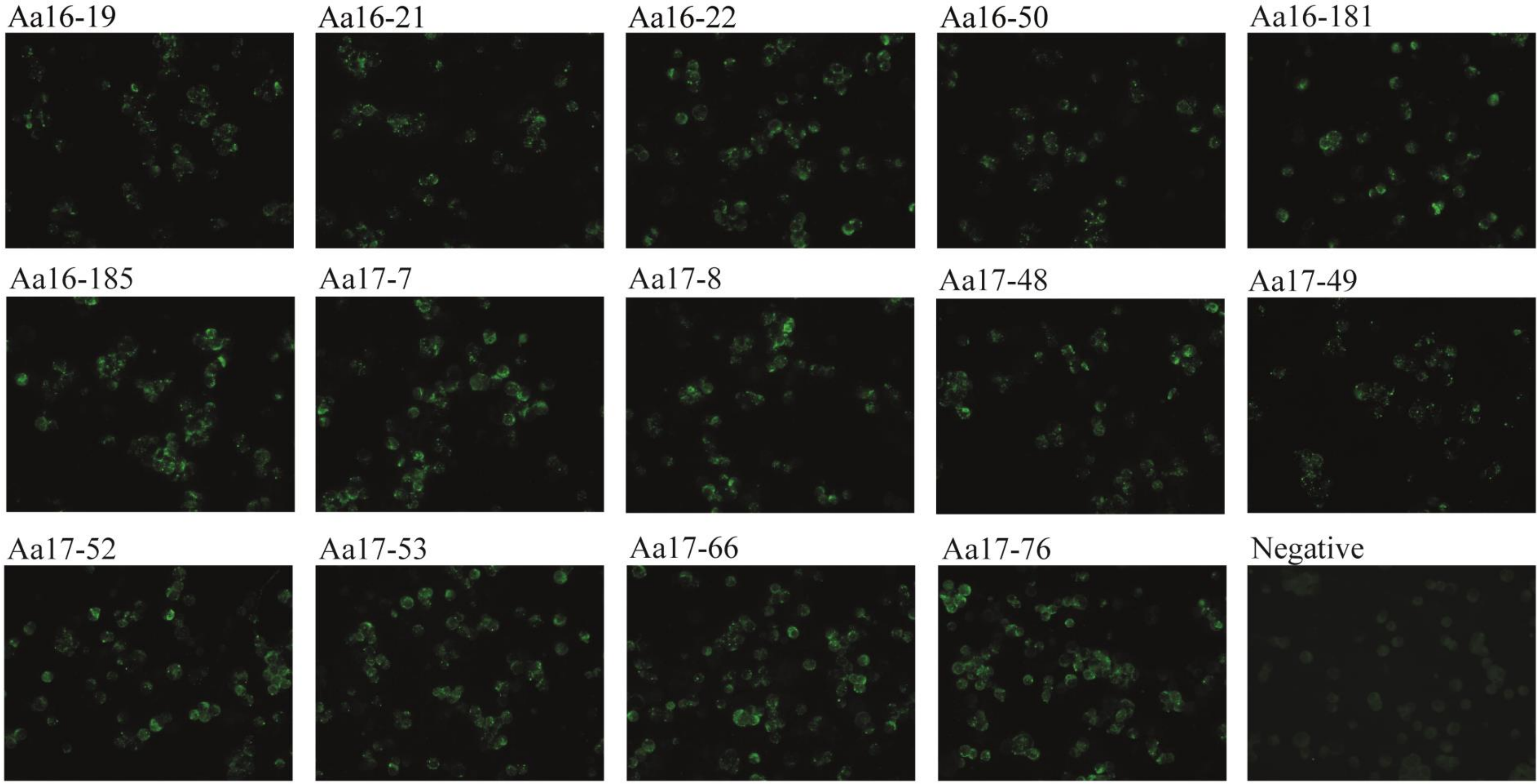

Supplementary Fig 2.

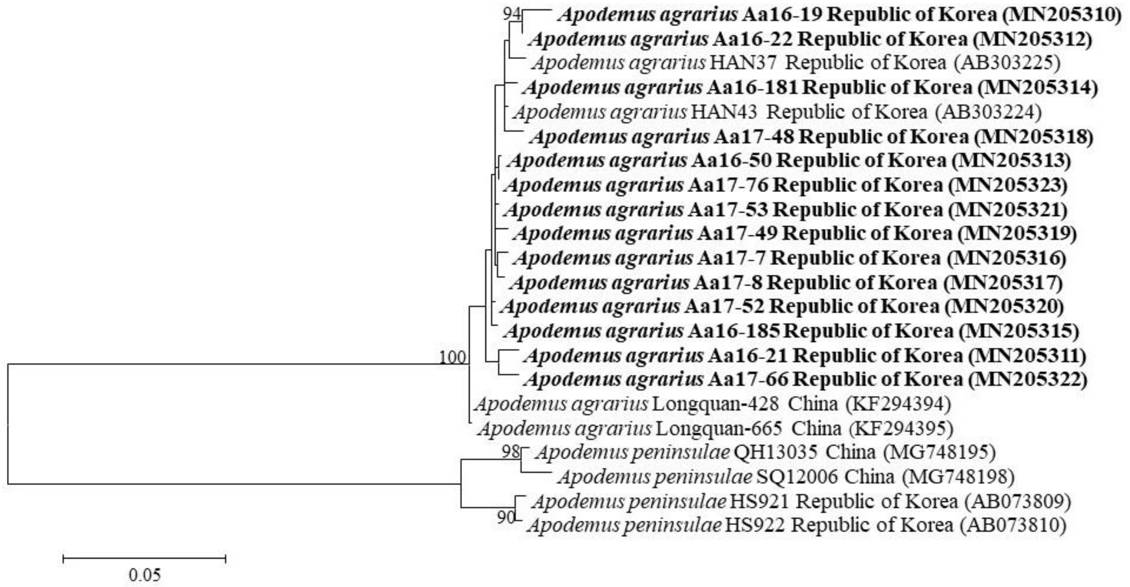

Supplementary Fig 3.

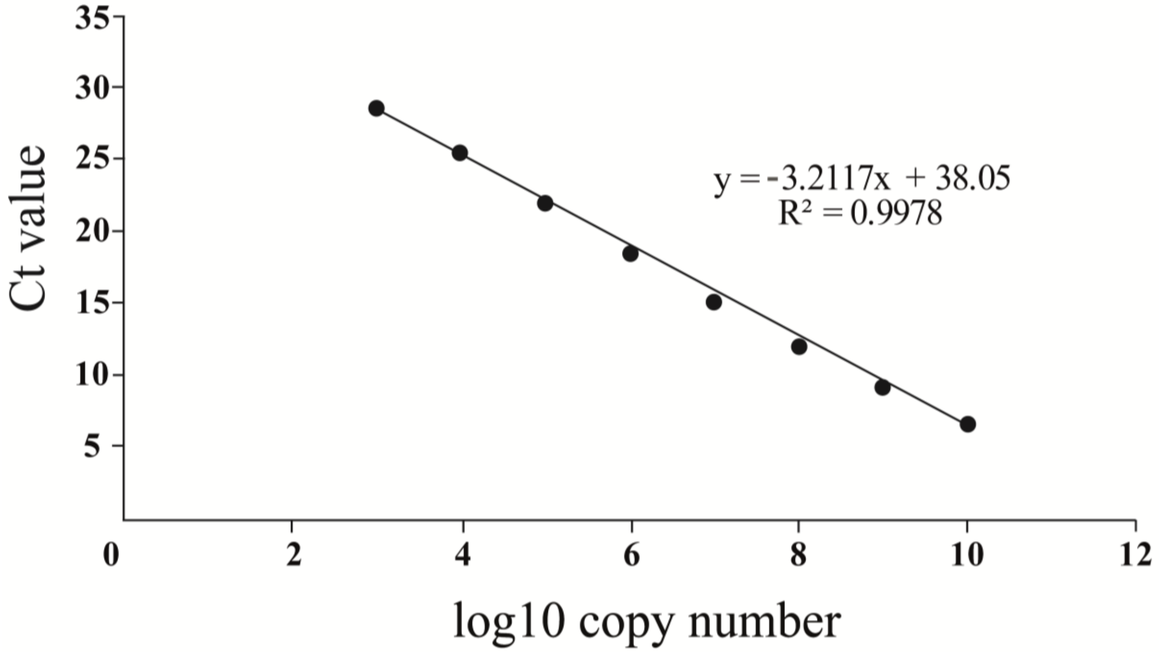

Supplementary Table 1. Summary of total reads and read mapping to Hantaan orthohantavirus (HTNV) reference genome by SISPA NGS

Viral RNA copy number	Strain	Total reads	Reads mapped to reference/Total reads (%)	L segment		M segment		S segment	
				Reads mapped to reference	Depth of coverage[a]	Reads mapped to reference	Depth of coverage[a]	Reads mapped to reference	Depth of coverage[a]
10^5	Aa16-19	1,095,872	0.004	10	0.2	15	0.5	17	1.4
	Aa16-50	741,276	0.007	5	0.1	14	0.5	30	2.4
	Aa17-8	754,064	0.064	172	3.7	109	4.2	199	16.5
	Aa17-49	302,860	0.124	106	2.2	72	2.8	198	16.4
	**Average (%)[b]**	**723,518**	**0.033**	**73**	**2**	**53**	**2**	**111**	**9**
10^3 to 10^4	Aa16-181	657,936	0.003	7	0.1	2	0	8	0.6
	Aa16-185	1,044,134	0	0	0	0	0	4	0.3
	Aa17-48	1,303,396	0.003	12	0.2	12	0.4	9	0.7
	Aa17-52	297,316	0	0	0	0	0	0	0
	Aa17-53	378,488	0	0	0	0	0	0	0
	**Average (%)[b]**	**736,254**	**0.001**	**4**	**0**	**3**	**0**	**4**	**0**
10^2	Aa16-21	457,420	0	0	0	0	0	0	0
	Aa16-22	723,242	0	0	0	0	0	0	0
	Aa17-7	982,292	0	0	0	0	0	0	0
	Aa17-66	323,342	0	0	0	0	0	0	0
	Aa17-76	372,012	0	0	0	0	0	0	0
	**Average (%)[b]**	**571,661.6**	**0**	**0**	**0**	**0**	**0**	**0**	**0**
**Total**	**Average (%)[b]**	**673,832**	**0.011**	**22 (0.003%)**	**0**	**16 (0.002%)**	**1**	**33 (0.005%)**	**3**

- Aa, *Apodemus agrarius*

[a]Depth of coverage was calculated by the number of mapped reads (read length × number of reads matching to the reference/reference genome size).

[b]Average percentage rate was calculated by dividing the Reads mapped to reference over the total reads.

Supplementary Table 2. Summary of total reads and read mapping to Hantaan orthohantavirus (HTNV) reference genome by target capture NGS

Viral RNA copy number	Strain	Total reads	Reads mapped to reference / Total reads (%)	L segment		M segment		S segment	
				Reads mapped to reference	Depth of coverage[a]	Reads mapped to reference	Depth of coverage[a]	Reads mapped to reference	Depth of coverage[a]
10^5	Aa16-19	2,051,894	98.7	554,438	11,966.3	831,133	32,408.7	639,667	53,179.9
	Aa16-50	1,159,098	87.7	378,331	8,165.4	282,303	11,007.9	355,732	29,574.4
	Aa17-8	3,079,028	96.8	846,407	18,267.8	944,794	36,840.7	1,190,733	98,993.7
	Aa17-49	13,908,190	99.3	4,228,824	91,269.6	4,388,562	171,124.8	5,191,048	431,567.1
	**Average (%)[b]**	**5,049,553**	**98.2**	**1,502,000 (29.7%)**	**32,417.4**	**1,611,698 (31.9%)**	**62,846**	**1,844,295 (36.5%)**	**153,329**
10^3 to 10^4	Aa16-181	144,422	62.9	30,350	655	21,890	853.6	38,536	3,203.8
	Aa16-185	305,190	79.6	58,358	1,259.5	94,914	3,701	89,659	7,454
	Aa17-48	470,938	96.3	98,582	2,127.7	163,740	6,384.8	191,391	15,911.6
	Aa17-52	70,062	14.9	3,710	80.1	1,089	42.5	5,633	468.3
	Aa17-53	95,632	36.5	9,634	207.9	3,128	122	22,111	1,838.2
	**Average (%)[b]**	**217,249**	**76.7**	**40,127 (18.5%)**	**866**	**56,952 (26.2%)**	**2,221**	**69,466 (32.0%)**	**5,775**
10^2	Aa16-21	6,486	28.5	573	12.4	309	12	969	80.6
	Aa16-22	23,936	22.0	1,650	35.6	1,600	62.4	2,023	168.2
	Aa17-7	2,256,662	0.017	102	2.2	88	3.4	178	14.8
	Aa17-66	49,312	4.3	526	11.4	28	1.1	1,565	130.1
	Aa17-76	69,442	2.8	742	16	38	1.5	1,168	97.1
	**Average (%)[b]**	**481,168**	**0.48**	**719 (0.2%)**	**16**	**413 (0.1%)**	**16**	**1,181 (0.3%)**	**98**
**Total**	**Average (%)[b]**	**1,692,164**	**87.3**	**443,731 (26.2%)**	**9,577**	**480,973 (28.4%)**	**18,755**	**552,172 (32.6%)**	**45,906**

- Aa, *Apodemus agrarius*

[a]Depth of coverage was calculated by the number of mapped reads (read length × number of reads matching to the reference/reference genome size).

[b]Average percentage rate was calculated by dividing the Reads mapped to reference over the total reads.

Supplementary Table 3. Summary of total reads and read mapping to Hantaan orthohantavirus (HTNV) reference genome by amplicon NGS

Viral RNA copy number	Strain	Total reads	Reads mapped to reference / Total reads (%)	L segment		M segment		S segment	
				Reads mapped to reference	Depth of coverage[a]	Reads mapped to reference	Depth of coverage[a]	Reads mapped to reference	Depth of coverage[a]
10^5	Aa16-19	526,302	93.9	172,464	3,226.5	199,462	6,288.5	122,066	9,683.8
	Aa16-50	816,926	98.8	273,009	5,354	309,976	9,982.2	224,153	17,120.5
	Aa17-8	1,685,526	99.4	517,050	10,730	632,468	20,213.7	525,785	39,899.6
	Aa17-49	1,667,600	99.5	569,097	11,237	595,947	19,096.4	494,627	37,093.2
	**Average (%)[b]**	**1,174,089**	**98.7**	**382,905 (32.6%)**	**7,637**	**434,463 (37.0%)**	**13,895**	**341,658 (29.1%)**	**25,949**
10^3 to 10^4	Aa16-181	585,162	98.9	195,109	3,853.8	228,633	7,307.4	154,869	11,223.4
	Aa16-185	1,306,312	98.7	414,241	8,046.6	531,116	16,670.9	344,268	24,947.8
	Aa17-48	5,918,892	99.6	1,987,812	38,770	2,072,442	67,227.8	1,837,304	137,902.4
	Aa17-52	1,501,262	95.8	391,026	7,525.6	471,629	15,259.5	574,964	42,586.3
	Aa17-53	1,405,480	97.4	473,574	9,153.4	443,627	14,280.5	451,678	33,818
	**Average (%)[b]**	**2,143,422**	**98.7**	**692,352 (32.3%)**	**13,470**	**749,489 (35.0%)**	**24,149**	**672,617 (31.4%)**	**50,096**
10^2	Aa16-21	274,160	82.8	59,703	1,110.1	36,087	1,343.4	131,148	10,571.6
	Aa16-22	227,504	69.2	23,705	390.6	44,898	1,506.5	88,877	7,109.8
	Aa17-7	1,685,526	66.2	236,212	4,883.8	436,965	13,604.5	441,844	33,025.1
	Aa17-66	1,558,140	84.1	513,182	2,837.3	158,996	18,028.8	637,534	48,369.2
	Aa17-76	1,114,486	80.7	315,146	6,152.1	59,877	1,774.5	524,477	39,945.9
	**Average (%)[b]**	**971,963**	**76.3**	**229,590 (23.6%)**	**3,075**	**147,365 (15.2%)**	**7,252**	**364,776 (37.5%)**	**27,804**
**Total**	**Average (%)[b]**	**1,448,091**	**93.3**	**438,666 (30.3%)**	**8,091**	**444,437 (30.7%)**	**15,185**	**468,114 (32.3%)**	**35,235**

- Aa, *Apodemus agrarius*

[a]Depth of coverage was calculated by the number of mapped reads (read length × number of reads matching to the reference/reference genome size).

[b]Average percentage rate was calculated by dividing the reads mapped to reference over the total reads.

Supplementary Table 4. GenBank accession numbers of the Hantaan orthohantavirus (HTNV) sequences.

Strain	Target capture NGS			Amplicon NGS		
	L	M	S	L	M	S
Aa16-19	MK548691	MK548682	MK548673	MK548664	MK548655	MK548646
Aa16-50	MK548692	MK548683	MK548674	MK548667	MK548658	MK548649
Aa16-181	MK548693	MK548684	MK548675	MK548668	MK548659	MK548650
Aa16-185	MK548694	MK548685	MK548676	MK548669	MK548660	MK548651
Aa17-8	MK548695	MK548686	MK548677	MH598476	MH598490	MH598504
Aa17-48	MK548696	MK548687	MK548678	MH598477	MH598491	MH598505
Aa17-49	MK548697	MK548688	MK548679	MH598478	MH598492	MH598506
Aa17-52	MK548698	MK548689	MK548680	MK548670	MK548661	MK548652
Aa17-53	MK548699	MK548690	MK548681	MH598479	MH598493	MH598507
Aa16-21	-[a]	-	-	MK548665	MK548656	MK548647
Aa16-22	-	-	-	MK548666	MK548657	MK548648
Aa17-7	-	-	-	MH598475	MH598489	MH598503
Aa17-66	-	-	-	MK548671	MK548662	MK548653
Aa17-76	-	-	-	MK548672	MK548663	MK548654

[a]Viral genomic sequences were not obtained due to the low coverage.

Supplementary Table 5. SRA accession codes of sequencing data.

Strain	SISPA NGS	Target capture NGS	Amplicon NGS
Aa16-19	SRR10272892	SRR10272853	SRR10272579
Aa16-21	SRR10272893	SRR10272854	SRR10272580
Aa16-22	SRR10272894	SRR10272856	SRR10272581
Aa16-50	SRR10272896	SRR10272857	SRR10272625
Aa16-181	SRR10272897	SRR10272859	SRR10272626
Aa16-185	SRR10273084	SRR10272862	SRR10272627
Aa17-7	SRR10273105	SRR10272863	SRR10272628
Aa17-8	SRR10273085	SRR10272864	SRR10272803
Aa17-48	SRR10273108	SRR10272865	SRR10272817
Aa17-49	SRR10273107	SRR10272887	SRR10272828
Aa17-52	SRR10273103	SRR10272888	SRR10272829
Aa17-53	SRR10273104	SRR10272889	SRR10272830
Aa17-66	SRR10273106	SRR10272890	SRR10272843
Aa17-76	SRR10273086	SRR10272891	SRR10272844
